# Channel Allocation and Equity in Preventive Campaigns for Older Adults: Agent-Based Modeling Study

**DOI:** 10.2196/88429

**Published:** 2026-04-01

**Authors:** Jihye Lee, Juyoung Park, Yuna Kim, Duk-Jo Kong

**Affiliations:** 1 Seoul National University, Seoul, Republic of Korea; 2 Gwangju Institute of Science and Technology, 123 Cheomdangwagi-ro, Gwangju, Republic of Korea, 82 62 715 2934

**Keywords:** preventive campaigns, channel allocation, older adults, agent-based modeling, distributional equity, resource allocation, budget constraints, influenza vaccination, loss framing, strategic planning

## Abstract

**Background:**

Preventive campaigns for older adults must decide how to allocate limited resources across media channels. However, these channel allocation and budget decisions rarely use explicit criteria for distributional equity or structured strategic planning tools. Consequently, health systems may optimize average uptake while leaving large gaps across socioeconomic groups and media use profiles.

**Objective:**

This study aimed to develop and apply a data-driven agent-based model as a strategic planning tool for preventive campaigns targeting older adults, comparing channel allocation, personalization, and loss framing options under explicit budget and equity guardrails.

**Methods:**

We built an agent-based model calibrated to national survey data from South Korea on influenza vaccination and routine health screening among older adults (vaccination, N=2405; screening, N=2400). Fifteen prespecified campaign scenarios varied channel allocation across television, digital, and print media; budget intensity; 2 equity-focused personalization strategies; and graded loss framing. Primary outcomes were final adoption and time to adoption. Equity outcomes included the minimum class-level adoption and 90‐10 gap across latent classes. Each scenario was simulated over 12 monthly steps with 100 Monte Carlo replications. We conducted sensitivity analyses varying link functions and key social reinforcement parameters.

**Results:**

Personalization improved uptake and equity relative to the integrated baseline. In the vaccination model (N=2405), adoption increased from 91.2% (n=2193) to 93.3% (n=2244) and 94.6% (n=2275). Minimum class-level adoption increased from 86.8% to 90.3% and 90.9%. The 90‐10 gap narrowed from 5.7 to 4.5 and 4.7 percentage points. In the screening model (N=2400), adoption increased from 83.8% (n=2011) to 88.2% (n=2117) and 89.5% (n=2148). Minimum class-level adoption increased from 77.6% to 83.2% and 85.3%. The 90‐10 gap narrowed from 9.2 to 7.4 and 6.2 percentage points. Television-only strategies achieved high adoption but had less favorable equity profiles than personalization. High-budget strategies achieved high adoption but required higher total exposure. Stronger loss framing produced small, monotonic gains in adoption and shortened the time to adoption without worsening equity in the tested range. Scenario rankings were stable in sensitivity analyses.

**Conclusions:**

This agent-based modeling study illustrates how ex ante planning can improve preventive campaign design by comparing channel allocation and personalization options under explicit equity and budget criteria. For campaigns targeting older adults, equity-focused reweighting and class-tailored television-digital portfolios improved or preserved mean adoption while strengthening distributional equity under fixed budgets. In contrast, undifferentiated channel diversification without personalization offered a less favorable efficiency-equity trade-off. These findings support integrating explicit equity guardrails into early-stage channel allocation and prioritizing targeted personalization over simple channel diversification. Future work should validate these patterns in other populations and health systems and link simulated diffusion trajectories with observed exposure and engagement in real-world campaigns. It should also extend guardrail-based planning tools to organizational settings and multiyear decision contexts.

## Introduction

### Background

Preventive behaviors among older adults, such as seasonal influenza vaccination and routine health screening, remain uneven across socioeconomic strata despite long-standing equity goals [1-4]. Multiple analyses in Europe and the United Kingdom report persistent or widening gaps in influenza vaccination uptake among adults aged ≥60 years by deprivation and education [1,2]. These studies also describe comparable heterogeneity in cancer screening participation and outcomes across European Union member states [3,4]. These disparities persist even where national coverage has improved, suggesting that changes in supply or eligibility alone are insufficient to close equity gaps. Vaccination and screening are related but behaviorally distinct preventive targets in older adults. Annual influenza vaccination is often an episodic, clinician-recommended decision, whereas routine health screening requires multistep planning, navigation of organizational procedures, and higher levels of health and digital literacy.

Daily life and health care for older adults are increasingly mediated by digital channels such as online news, social media, messaging apps, and patient portals [5-7]. Systematic reviews show wide variation in digital literacy, skills, and confidence among older adults, with distinct connectivity levels across user segments and pronounced social gradients in eHealth literacy [5-7]. Emerging digital health research links this digital divide to inequalities in self-management, use of online health services, and broader health outcomes among older adults [8-10]. Older adults are therefore not a single audience but a set of heterogeneous segments occupying different media and support ecologies that can amplify or dampen campaign effects.

Recent equity-focused work also emphasizes behavioral and social drivers of vaccination and screening—beliefs, norms, practical barriers, and motivation—rather than structural access alone. The World Health Organization’s behavioral and social drivers of vaccination framework and scoping reviews of influenza vaccination in older adults highlight that groups with fewer resources are often missing from the evidence base and that social context strongly shapes uptake [11,12]. For campaign planners, this implies that channel choice, sequencing, and audience segmentation should consider not only reach but also how media exposure interacts with social reinforcement and practical constraints in different subgroups. These challenges point to the need for explicit digital health strategic planning tools to guide channel allocation, personalization, and the use of equity guardrails in preventive campaigns for older adults.

### Current Evidence

#### Planning and Equity Frameworks

Implementation science models such as the PRECEDE-PROCEED model and the Reach, Effectiveness, Adoption, Implementation and Maintenance (RE-AIM)/Practical Robust Implementation and Sustainability Model (PRISM) have strengthened the understanding of reach, adoption, and sustainability in public health programs [13,14]. These models are increasingly applied to plan and evaluate interventions with an explicit focus on equity [15,16]. In parallel, equity frameworks such as PROGRESS-Plus (place of residence; race, ethnicity, culture, and language; occupation; gender and sex; religion; education; socioeconomic status; and social capital—plus additional equity-relevant factors) and related disparity indices are widely used to structure and report the distributional impacts of public health interventions in systematic reviews and policy analyses [15].

However, in most applications, these tools are applied after interventions have been implemented. Most studies focus on describing who was reached and how effects varied [1,13,17]. They rarely generate prospective allocation rules across channels or segments under budget and equity constraints. Even less often are they operationalized as ex ante decision support tools for channel portfolios.

#### Digital and Social Media Campaigns

Systematic reviews of digital and social media campaigns show complex exposure pathways and small but generally positive average effects on health behaviors, with substantial heterogeneity across platforms, populations, and outcomes [18-20]. However, most evaluations rely on intermediate metrics—impressions, clicks, likes, or other low-level engagement—rather than behavior change [18-20]. This reliance on proxy outcomes complicates the assessment of the added value of specific channels beyond offline activities.

As a result, planners have limited operational guidance on how to allocate budgets across channels. Key decisions include whether to concentrate resources on a single high-leverage channel (eg, television), distribute spending across multiple platforms, or adjust the mix over time as part of strategic planning.

#### Personalization and Equity

Personalization and targeting are widely promoted in digital health to improve relevance and engagement, including for older adults [21,22]. Conceptual and empirical reviews describe approaches ranging from simple demographic tailoring to rule-based and data-driven personalization, but their equity implications are mixed and often underdocumented [21,23]. A recurring concern is that personalization may disproportionately benefit digitally skilled or socially advantaged groups, thereby widening disparities [23,24].

Although equity-oriented frameworks encourage inclusive targeting and prioritization of underserved groups [25], there is little mechanistic evidence comparing fundamentally different personalization philosophies. Such contrasts include cautious, vulnerability-focused adjustments versus segment-optimized channel mixes evaluated under common efficiency and equity metrics. These contrasts suggest that the same personalization or channel strategy could plausibly yield different efficiency-equity profiles for vaccination and screening. However, existing planning models rarely examine these targets side by side within a unified framework.

#### Equity Metrics as Planning Tools

Equity metrics and frameworks have also advanced. Entropy-based indices such as Theil-type measures summarize the distribution of health outcomes across social strata and capture both between- and within-group variation [26]. In parallel, PROGRESS-Plus has become a standard for conceptualizing equity dimensions in systematic reviews and equity-relevant evaluations [25].

In many applications, equity metrics are used post hoc to describe which groups benefited [25,26]. Researchers have proposed more prescriptive approaches that incorporate distributional objectives into economic evaluation, including distributional cost-effectiveness analysis and pragmatic aggregate variants [27]. However, these methods remain uncommon in routine resource allocation practice [27].

#### Network and Agent-Based Modeling

Network and simulation approaches, especially agent-based models (ABMs), represent individuals as interacting agents linked through online and offline networks [28,29]. These models can explicitly capture heterogeneous exposure, social influence, and timing. Reviews describe ABMs as “policy laboratories” that allow planners to test alternative portfolios and explore system behavior before real-world deployment [28,29]. Network experiments and diffusion studies further show that many protective behaviors spread as complex contagions that require repeated, multisource reinforcement [30].

Despite this potential, most ABM applications in public health focus on infectious disease transmission or generic opinion dynamics [28]. Only a small but growing set of studies uses ABMs to examine vaccination strategies or health communication interventions [31,32]. Even in this work, models rarely consider resource constraints, channel portfolios, and equity-focused objectives for older adult vaccination or screening within a single, reproducible framework [31,32]. These gaps motivate data-driven ABM approaches that can support strategic planning for older adult vaccination and screening campaigns under explicit equity guardrails.

#### Research Gaps and Study Objectives

Against this backdrop, several gaps remain at the intersection of digital health campaigns, personalization, and equity for older adults:

- Budget-constrained channel portfolios: Despite detailed descriptions of older adults’ digital heterogeneity and the digital divide, we found no models that explicitly link these patterns to budget-constrained channel portfolios [5-10,21,22]. Such models would show how much to invest in television, digital, or print media over time for preventive campaigns in this population.
- Contrasting personalization philosophies: The common assumption that personalization improves efficiency but threatens equity has not been tested in mechanistic models. In particular, few models compare fundamentally different personalization strategies—for example, a cautious, outcome-targeted boost for the worst-off class versus a fully segment-optimized, media affinity–based allocation—under common outcome and equity metrics.
- Target-specific behavioral profiles: Existing planning models seldom account for the fact that vaccination and screening differ in baseline uptake, decision complexity, and system dependencies in older adults. Few studies evaluate whether a given channel or personalization strategy traces the same efficiency-equity trade-offs across these 2 targets.
- Equity metrics as guardrails: Existing equity metrics and frameworks are rarely embedded as binding constraints in campaign planning [11-13,15,24]. There is limited empirical work on how explicit guardrails (eg, minimum subgroup adoption, caps on adoption gaps) reshape the space of admissible channel and personalization strategies [11-13,15,24].
- Reproducible data-driven ABM for strategic planning: Most studies evaluate isolated interventions or channels. Few use reproducible, survey-calibrated ABMs that tie nationally representative data on media use, digital skills, social connectedness, and preventive behaviors to multichannel campaign scenarios [31,32]. There is particularly little work on “virtual laboratories” that vary channel mix, personalization, framing, and equity guardrails jointly under fixed budgets within a unified, budget-constrained planning framework [31,32].

**Figure 1.**
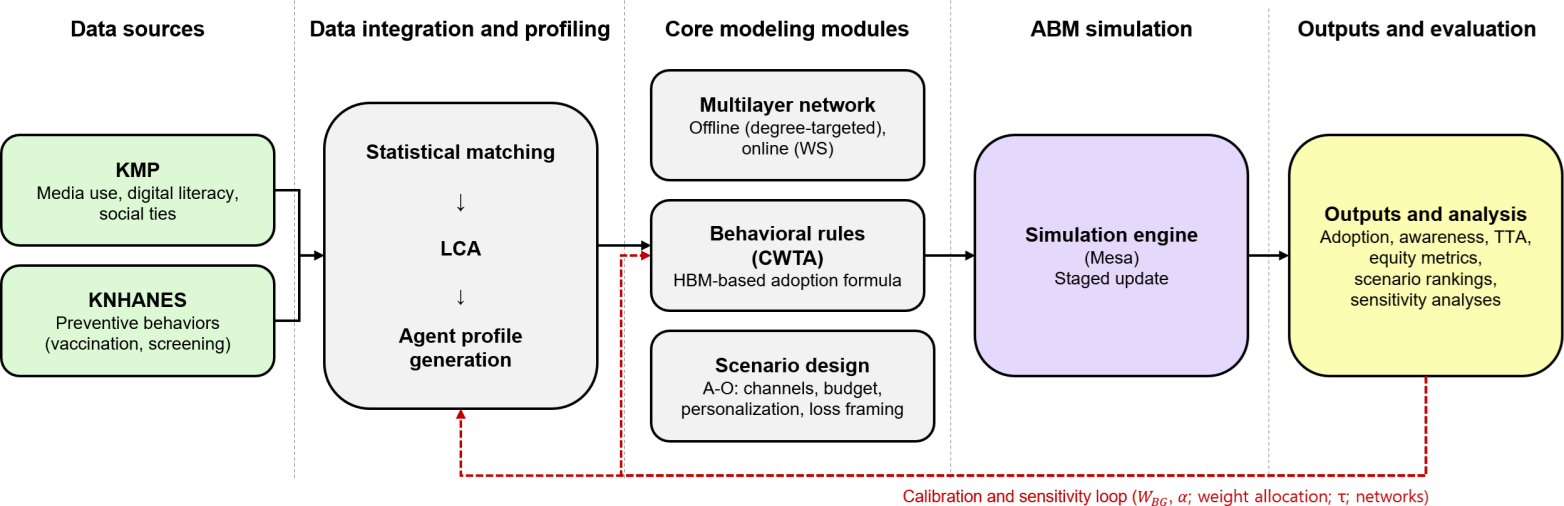
Overview of the data-to-ABM pipeline for evaluating channel allocation and personalization strategies under budget and equity guardrails. ABM, agent-based model; CWTA, cumulative weighted threshold adoption; HBM, health belief model; KMP, Korea Media Panel Survey; KNHANES, Korea National Health and Nutrition Examination Survey; LCA, latent class analysis; TTA, time to adoption; W_BG_, background exposure weight; WS, Watts-Strogatz small-world network.

This study addresses these gaps by developing a reproducible data-to-ABM pipeline for preventive campaigns for older adults. We focused on older adults in South Korea—a high-income, aging health system—and aim to derive strategic planning principles that may be adaptable to similar contexts. Using matched national survey data, we constructed a heterogeneous older adult population segmented by latent class, embedded agents in multilayer offline-online networks, and simulated 15 prespecified campaign scenarios under fixed budgets and explicit equity guardrails. Our goal was to provide a strategic planning environment that allows planners to compare channel mixes, personalization philosophies, and loss framing intensities under explicit equity guardrails and interpret the resulting portfolios as decision-support inputs for national or regional planning. Figure 1 summarizes the data-to-ABM pipeline from data sources to simulation outputs. It highlights data integration and profiling; core modeling modules; the simulation engine; and the calibration and sensitivity loop used to evaluate channel allocation, personalization, and loss framing under explicit equity guardrails.

We focused on 5 research questions (RQs):

RQ1: how do single-channel campaigns compare with a background-only baseline?

RQ2: do multichannel mixes produce synergy or dilution under fixed budgets?

RQ3: how do 2 contrasting personalization strategies—outcome-targeted (cautious equity-first) versus class-customized (segment-optimized)—affect mean outcomes?

RQ4: how do these personalization strategies reshape distributional equity across latent classes?

RQ5: does loss-framed messaging act as a secondary tuner of adoption, and do its effects differ between vaccination and screening?

## Methods

### Study Design

Campaign planning for older adults must account for how exposure unfolds over time and spreads through offline and online social ties [18,33-35]. These dynamics are difficult to represent in static regression or compartmental models, which assume homogeneous exposure and ignore network reinforcement [36,37]. The challenge is amplified by having 2 preventive targets with distinct determinants: influenza vaccination and routine screening.

To address these limitations, we developed an ABM that represents each older adult as an agent embedded in multilayer networks [38]. The model captures heterogeneous exposure, repeated social reinforcement, and channel-specific media effects under fixed campaign budgets. It enables prospective comparison of prespecified channel mixes, personalization strategies, and equity guardrails as alternative campaign designs. Therefore, we used the model as a strategic planning tool for digital health campaigns targeting older adults.

### Data Sources and Agent Construction

#### Data Sources and Statistical Matching

Two national surveys were combined: the 2022 Korea Media Panel Survey (KMP) [39] and the 2022 Korea National Health and Nutrition Examination Survey (KNHANES) [40]. KMP captured media use, digital literacy, and social relationship patterns, while KNHANES reported preventive behaviors including influenza vaccination and screening. Linking the 2 datasets enabled individual-level integration of media exposure, digital access, social connectedness, and preventive behaviors.

We restricted KNHANES recipients to adults aged ≥65 years and defined the KMP donor pool as adults aged ≥55 years to improve age comparability during matching. After harmonizing overlapping variables, we performed one-to-one nearest-neighbor matching with replacement using Gower distance on key sociodemographic covariates [41]. This approach assigned media, digital, and social attributes from KMP donors to matched KNHANES recipients while retaining KNHANES health outcomes. The matching diagnostics met our prespecified acceptance criterion; the full covariate list and diagnostics are reported in Multimedia Appendix 1.

#### Latent Class Analysis

Latent class analysis was applied to the matched dataset to identify classes of older adults with distinct media, digital, social, and preventive profiles [42]. The indicator domains included sociodemographics, digital literacy, media use, social activity, and vaccination and screening behaviors. Models with 3‐10 classes were evaluated using the Akaike information criterion (AIC), the Bayesian information criterion (BIC), entropy, and the bootstrap likelihood ratio test (K vs K–1). A 6-class solution was selected based on the lowest BIC and high entropy (BIC=86,578.57; entropy=0.92). Solutions with additional classes yielded marginal AIC improvements. However, they increased complexity without BIC gains. Accordingly, the 6-class structure was selected as the basis for class-based personalization in the simulation. Variable definitions, discretization rules, and full model comparison results are provided in Table S1 in Multimedia Appendix 1, and detailed class profiles are reported in Table S3 in Multimedia Appendix 2. For descriptive summaries, individuals were assigned to their modal class. For simulation inputs, posterior class probabilities were retained as personalization weights. This approach preserves interpretability and propagates classification uncertainty into the inputs. We treated class membership as time invariant over the 12-month simulation horizon.

#### Agent Initialization and Network Priors

Each matched individual was instantiated as an agent with a channel affinity vector and an offline degree prior derived from survey responses. Channel affinities captured digital and traditional media use. Offline degree priors were based on self-reported social activity frequency and served as individual target degrees during offline network construction. Details of affinity construction, degree-level mapping, and multilayer network generation are provided in Multimedia Appendix 1 and in the Network and State Dynamics section.

### Outcomes and Equity Metrics

Primary outcomes were final adoption at month 12 (proportion of agents who adopted) and mean time to adoption among adopters. We used “adoption” as a generic term for vaccination uptake and screening participation. Awareness was treated as a supporting outcome.

We assessed equity across latent classes using 5 distributional metrics. A_min_ denoted the minimum class-level adoption. We defined the 90‐10 gap as the difference between the 90th and 10th percentiles of class-level adoption and relative disparity index (RDI) as the ratio of maximum to minimum class-level adoption. We also calculated 2 entropy-based indices, Theil T and Atkinson A_0.5_ (*ε*=0.5), as descriptive measures of inequality [43,44]. For interpretation, we used prespecified illustrative planning benchmarks (“equity guardrails”): A_min_≥0.60, 90‐10 gap≤0.25, and RDI≤1.5. These thresholds are tunable planning benchmarks for comparative portfolio assessment and not universal ethical or empirical standards. In practical terms, they set (1) a minimum-adoption floor for the lowest-adoption class and (2) limits on absolute and relative disparities. Specifically, we required ≤25 percentage points for the 90‐10 gap and a ratio ≤1.5 for RDI. These thresholds use the same probability scale as the calibrated uptake in our setting. Theil T and Atkinson A_0.5_ were reported descriptively and were not used for guardrail screening.

Scenario-level adoption and equity metrics for all campaign scenarios (A-O), for both vaccination and screening, are reported in Tables S1 and S2 in Multimedia Appendix 2. We ran 100 Monte Carlo replications per scenario. We report all outcomes as means with 95% percentile intervals (2.5th-97.5th percentile), stratified by task (vaccination vs screening).

### Scenario Design

#### Channel Allocation and Budget (RQ1-RQ2)

We evaluated 15 prespecified scenarios (A-O) that varied in 4 design factors: channel allocation, budget intensity, class-level personalization, and loss framing. Exposure weights were specified as relative per-step exposure intensity parameters (rather than monetary spending). Scenarios A to H tested single channels or fixed-sum channel mixes at a common per-step media budget. Scenario A served as a control with no paid media exposure; awareness could arise from background exposure and social awareness. Scenarios B to D isolated individual channels (television [TV], digital, or print). Scenarios E to H implemented fixed-sum mixes combining 2 or 3 channels.

Scenarios I and J varied total budget intensity while keeping channel shares fixed. Scenario I scaled down exposure relative to the fixed-sum TV + digital mix, whereas scenario J scaled it up. Together, these scenarios represented low- and high-budget variants without changing the underlying channel mix.

#### Personalization: Efficiency and Equity (RQ3-RQ4)

Scenarios K and L introduced class-level personalization based on latent classes. Scenario K implemented equity-focused reweighting. For each task, the lowest-baseline uptake class received a small increase in television or digital exposure. All other classes had TV + digital exposure reduced by equal margins to maintain a constant class-average TV + digital budget. This design represents a conservative, budget-neutral policy that nudges media toward the lowest-baseline uptake class.

Scenario L assigned class-specific television and digital weights by latent class under the same class-level budget parity. Weights were tailored to each class’s baseline media use profile (television-heavy, digital-heavy, or mixed), rather than applying a uniform mix. This strategy represents a more aggressive, class-tailored policy that explicitly aligns channel mix with each class’s baseline media use profile.

#### Message Framing (RQ5)

Scenarios M to O applied loss framing multipliers (k_neg_) to television and/or digital exposure. Meta-analytic evidence suggests that framing effects are generally modest and behavior dependent. Gain framing shows advantages for prevention behaviors, whereas effects are mixed or weak for detection behaviors [45,46]. Accordingly, we tested k_neg_ values near 1.0, that is, 0.95 to –1.10 for vaccination and 1.05 to 1.20 for screening [45,46]. Tables1  2 report the exact channel weights and multiplier values for all scenarios.

**Table 1.** Channel allocation weights for campaign scenarios A to L.

Scenario	Name	Purpose	W_TV^c^_	W_DG^c^_	W_Print^c^_
A^a^	Control	Background only	0.000	0.000	0.000
B	TV^b^-only	Isolate TV	0.100	0.000	0.000
C	Digital-only	Isolate digital	0.000	0.100	0.000
D	Print-only	Isolate print	0.000	0.000	0.100
E	TV + digital	Fixed-sum mix	0.050	0.050	0.000
F	TV + print	Fixed-sum mix	0.050	0.000	0.050
G	Digital + print	Fixed-sum mix	0.000	0.050	0.050
H	All channels	Integrated mix	0.033	0.033	0.033
I	Low-budget	Scaled-down exposure	0.025	0.025	0.000
J^d^	High-budget	Scaled-up exposure	0.200	0.200	0.000
K^e^	Equity-focused reweighting	Reweight toward lowest-baseline class	Class dependent	Class dependent	0.000
L^f^	Class-tailored channel portfolios	Class-tailored channel mix	Class dependent	Class dependent	0.000

a Background-only awareness with per-step spillover (W_BG_=0.12 for vaccination, 0.10 for screening). W_BG_ denotes background exposure weight.

b TV: television.

c W_TV_, W_DG_, and W_Print_ denote per-step channel exposure weights for television, digital, and print media, respectively. Weights sum to approximately 0.10 (minor differences reflect rounding).

d Scenario J increases the total exposure (budget intensity) while keeping the channel shares fixed.

e Scenario K varies the intensity of the equity-focused reweighting (δ ∈ {0.02, 0.05}). For vaccination, the lowest-baseline uptake class was Class 6 (W_TV_=0.00, W_DG_ = 0.10 + δ); for screening, the lowest-baseline uptake class was Class 1 (W_TV_ = 0.10 + δ, W_DG_=0.00). In both cases, all other classes received symmetric low-intensity weights (W_TV_ = 0.05 − δ/10; W_DG_= 0.05 − δ/10). Results for *δ*=0.02 are reported in the main text, and *δ*=0.05 is presented as a higher-intensity sensitivity variant in Table S2 in Multimedia Appendix 3.

f Weights vary by latent class: classes 1 and 5: W_TV_=0.10; class 6: W_DG_=0.10; classes 2‐4: W_TV_=0.05 and W_DG_=0.05.

**Table 2.** Loss framing scenarios M to O with intensity multiplier ranges.^a^

Scenario	Name	Definition	W_TV^d^_	W_DG^d^_	W_Print^d^_
M	TV^b^ (loss framing)	B with W_TV_ × k_neg^c^_	0.10 × k_neg_	0.00	0.00
N	Digital (loss framing)	C with W_DG_ × k_neg_	0.00	0.10 × k_neg_	0.00
O	TV + digital (loss framing)	E with W_TV_/W_DG_ × k_neg_	0.05 × k_neg_	0.05 × k_neg_	0.00

a Vaccination: {0.95, 1.00, 1.05, 1.10}; Screening: {1.05, 1.10, 1.15, 1.20}. All exposure probabilities were clipped to [0,1].

b TV: television.

c k_neg_ denotes the loss framing intensity multiplier applied to baseline channel weights.

d W_TV_, W_DG_, and W_Print_ denote per-step channel exposure weights for television, digital, and print media, respectively. The weights sum to approximately 0.10 (minor differences reflect rounding).

### Network and State Dynamics

#### State Dynamics

The simulation ran over 12 monthly steps (t=1, …, 12). At each step, we iterated agents in random order and applied state conditional rules. At each time step, we first updated awareness and then updated adoption conditional on awareness. Once adopted, agents remained in the adopted state (state=2).

At each time step t, the awareness probability combined 3 components. The first component was background exposure. The second was campaign media exposure, determined by scenario-specific channel weights and agent-level channel use for television, digital, and print media. The third was the share of aware neighbors, weighted by ωa. In the control scenario A, all paid media terms were set to zero, and awareness arose from background exposure and social awareness (spillover).

Conditional on awareness, the adoption probability combined baseline propensity, media exposure, and social reinforcement. The reinforcement term activated only when at least 2 neighbors had adopted (threshold *τ*=2) and influenced adoption but not awareness. In loss framing scenarios M to O, we multiplied the television and digital exposure terms by k_neg_. All probabilities were clipped to the [0,1] interval. Textbox 1 provides a brief road map of the model logic (awareness, adoption, and reinforcement) and how to read the simulation outputs.

Textbox 1. **How the model works and how to read the outputs.**

1. What the model simulates: the model simulates how campaign exposure and social ties translate into adoption over 12 monthly steps.
2. Awareness (notice the message): an agent becomes aware via background exposure, paid media (scenario weights × the agent’s channel use), and social awareness (neighbors who are aware).
3. Adoption (take action): only if aware, the agent may adopt based on baseline propensity plus media and social influence.
4. Reinforcement (social proof): social influence applies only after at least *τ*=2 neighbors have already adopted.
5. Once adopted, always adopted: adoption is an absorbing state through month 12.
6. Outputs (how to read results): scenarios will be compared on final adoption, time to adoption, and equity across classes (A_min_, 90‐10 gap, relative disparity index [RDI]); the results will be interpreted as relative trade-offs under the same constraints and not as point forecasts.

#### Contact Structure

We modeled contacts as a 2-layer network with offline and online ties. For social influence, each agent’s neighborhood was defined as the union of its offline and online neighbors.

For baseline and channel-mix scenarios (A-H), we generated the analytic agents (vaccination, N=2405; screening, N=2400) and both network layers once and held them fixed across all Monte Carlo replications. For intervention and framing scenarios (I-O), we regenerated both layers at the start of each replication to reflect structural uncertainty while preserving a stable baseline design. This design choice was used to (1) reduce Monte Carlo variance when comparing fixed-budget channel-mix scenarios (A-H) and (2) incorporate structural uncertainty for intervention or framing variants (I-O).

Offline social layer (face-to-face): we mapped survey-based social activity frequency to an agent-specific degree target. We then built an undirected graph using a degree-targeted algorithm while avoiding self-loops and multi-edges [47,48].

Online social layer (network-based): we constructed the online layer as a Watts-Strogatz small-world network with a mean degree of 6 and rewiring probability of 0.10 [49].

### Calibration and Internal Validation

We calibrated 2 parameters for each task to match KNHANES 2022 targets for older adults (vaccination uptake 0.848; screening participation 0.744) [40]. We varied the baseline propensity multiplier and the background exposure over prespecified ranges. We then selected the parameter pair that minimized the absolute deviation from these targets under the background-only control scenario (A). This procedure yielded the values α_vaccine_=1.15 with W_BG_=0.12 and α_screening_=1.03 with W_BG_=0.10.

We assessed calibration quality using target deviation summaries and decile reliability plots that compared survey targets with deciles of baseline propensity [50-52]. These diagnostics used individual-level outputs aggregated across Monte Carlo replications. Tables S1 and S2 in Multimedia Appendix 4 provide the best-fitting parameter sets and the per-class calibration summaries. In this study, “validation” refers to internal diagnostics (calibration-to-target and reliability checks) within a survey-calibrated framework and does not constitute external validation using real-world campaign data.

### Sensitivity Analyses

We assessed robustness along 4 prespecified axes: link function, weight allocation, the social reinforcement threshold, and network uncertainty. For each axis, we reestimated scenario-level adoption vectors under the corresponding sensitivity configuration and compared them with the baseline ordering. We quantified rank stability using Spearman correlations and maximum rank shifts (Δrank) and treated ρ≥0.90 and Δrank≤1 as heuristic benchmarks for high stability. Tables S1 and S2 in Multimedia Appendix 3 provides the full protocols, stability statistics, and intensity variants for the personalization strategy [53-55].

### Ethical Considerations

This study used deidentified, publicly available microdata (KMP 2022; KNHANES 2022) [39,40] with no direct participant contact. Consent to participate was not applicable. Under institutional policy for secondary analyses of deidentified public datasets, formal institutional review board approval was not required. Under article 2(1) of the Bioethics and Safety Act of Korea [56], a “human subjects research project” is defined as research involving direct interaction with individuals or the use of identifiable personal information. As this study used only deidentified secondary data and did not involve identifiable information, it does not fall under this definition. Furthermore, according to article 13(1) of the Enforcement Rule of the Bioethics and Safety Act of Korea [57], research using publicly available data or research that does not collect or record personally identifiable information may be exempt from institutional review board review.

## Results

### Sample and Class Overview

The matched analytic sample included 2405 adults aged ≥65 years. Latent class analysis identified 6 classes with distinct sociodemographic and media use profiles [42]. Class sizes ranged from 245 (10.2%) to 534 (22.2%).

Baseline uptake varied across classes; the class-specific n and N are reported in Table S3 in Multimedia Appendix 2. Class 6 had the lowest baseline vaccination uptake, and Class 1 had the lowest baseline screening participation.

### Calibration and Internal Validation

We calibrated the baseline parameters to match the empirical adoption targets for older adults in KNHANES 2022 [40]. The targets were 2039 of 2405 (84.8%) for vaccination and 1786 of 2400 (74.4%) for screening. The selected parameter sets matched these targets closely (absolute deviation≤0.002). Calibration metrics supported good fit: Brier scores were 0.142 for vaccination and 0.187 for screening; calibration slopes were 1.15 and 1.08, respectively; and intercepts were −0.22 and −0.08, respectively [50-52].

**Figure 2.**
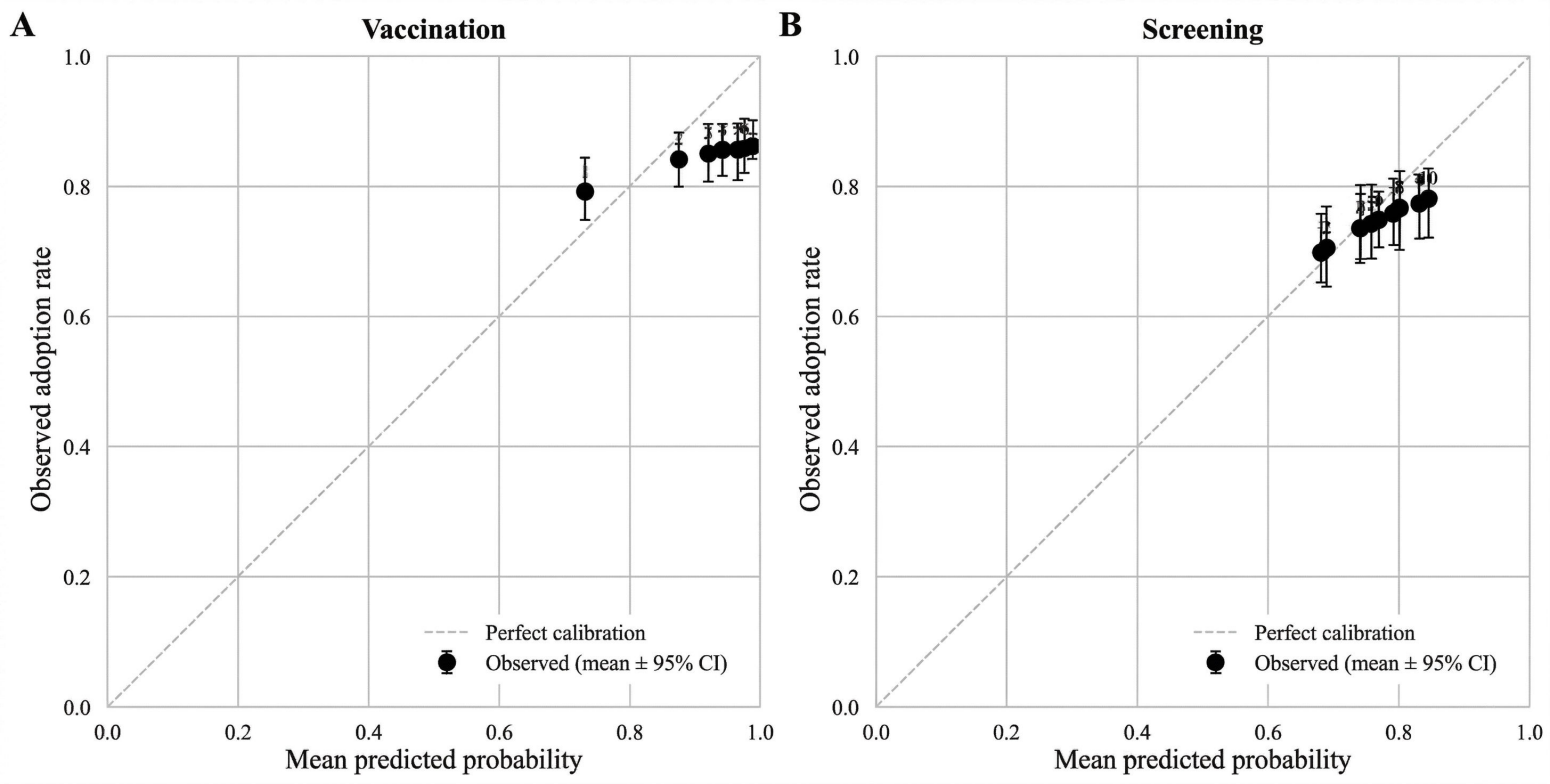
Decile reliability plots comparing mean predicted adoption probabilities with observed adoption rates for vaccination (A) and screening (B) under the calibrated background-only scenario.

Decile reliability plots under the calibrated background-only scenario showed close agreement between predicted and observed adoption probabilities (Figure 2) [50-52]. We analyzed 2405 individuals for vaccination and 2400 for screening (5 screening nonresponses excluded). Per-class calibration summaries are reported in Table S2 in Multimedia Appendix 4.

### Channel Allocation and Budget (RQ1-RQ2)

For RQ1, we compared each single-channel campaign (B-D) with the background-only baseline (A). The TV-only campaign (B) produced the largest gains. For vaccination (N=2405), final adoption increased from 85% (n=2044) in A to 94.3% (n=2268) in B (9.3 percentage points, 95% PI 7.3‐11.0). Mean time to adoption decreased from 6.18 (SD 0.07) to 5.34 (SD 0.06) months (0.84 months faster, 95% PI 0.65‐0.94). For screening (N=2400), adoption increased from 74.5% (n=1788) to 89.4% (n=2146; 14.9 percentage points, 95% PI 12.9‐17.0). Mean time to adoption decreased from 6.88 (SD 0.07) to 6.09 (SD 0.07) months (0.79 months faster, 95% PI 0.65‐0.94). Digital-only and print-only campaigns produced smaller gains. For vaccination, the digital-only and print-only campaigns increased adoption by about 139 adopters (5.8 percentage points) and about 17 adopters (0.7 percentage points), respectively. For screening, the corresponding gains were about 199 adopters (8.3 percentage points) and 29 adopters (1.2 percentage points).

**Figure 3.**
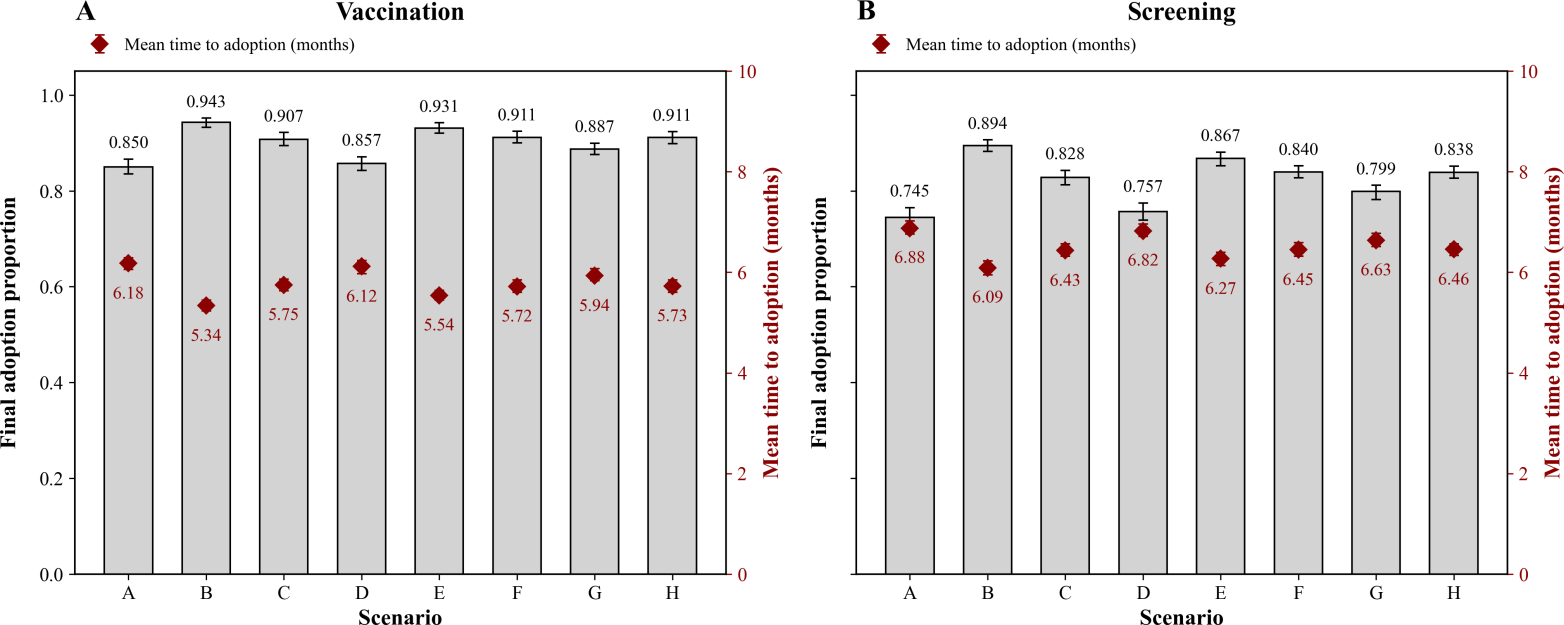
Final adoption and time-to-adoption outcomes across scenarios A to H for vaccination (A) and screening (B). Scenarios: A, control; B, television (TV)-only; C, digital-only; D, print-only; E, TV + digital; F, TV + print; G, digital + print; H, all channels; I, low-budget; J, high-budget; K, equity-focused reweighting; L, class-tailored channel portfolios.

For RQ2, we held the per-step media budget constant and compared mixed-channel strategies (E-H) with the TV-only benchmark (B). All mixed strategies (TV + digital, TV + print, digital + print, and all channels) improved upon the background-only baseline for both vaccination and screening; however, none surpassed the TV-only campaign in either final adoption or time to adoption (Figure 3). For the TV + digital mix (E), the synergy index (difference in adoption between the mixed strategy and the best single-channel strategy) was slightly negative: −1.2 percentage points (95% PI −3.6 to 1.2 percentage points) for vaccination and −2.3 percentage points (95% PI −5.8 to 1.3 percentage points) for screening, corresponding to a dilution of roughly 1 to 2 percentage points relative to the best single-channel option. Other mixed strategies showed similarly nonpositive synergy. Tables S1 and S2 in Multimedia Appendix 2 provide full scenario-level adoption and equity metrics for all campaigns (A-O).

### Personalization: Efficiency and Equity (RQ3-RQ4)

We compared the integrated baseline (H) with 2 personalization strategies: equity-focused reweighting (K) and class-tailored channel portfolios (L). For vaccination (N=2405), mean adoption increased from 91.2% (n=2193) in H to 93.3% (n=2244) in K and 94.6% (n=2275) in L. Furthermore, A_min_ increased from 86.8% (H) to 90.3% (K) and 90.9% (L). The 90‐10 gap narrowed from 5.7 percentage points in H to 4.5 and 4.7 percentage points in K and L, respectively. For screening (N=2400), mean adoption increased from 83.8% (n=2011) in H to 88.2% (n=2117) in K and 89.5% (n=2148) in L. Moreover, A_min_ increased from 77.6% (H) to 83.2% (K) and 85.3% (L). The 90‐10 gap narrowed from 9.2 percentage points to 7.4 and 6.2 percentage points in K and L, respectively.

Across both targets, strategy H had the lowest mean adoption and A_min_. Strategy L achieved the highest mean adoption and A_min_, and both personalization strategies (K and L) reduced the between-class disparities relative to H.

**Figure 4.**
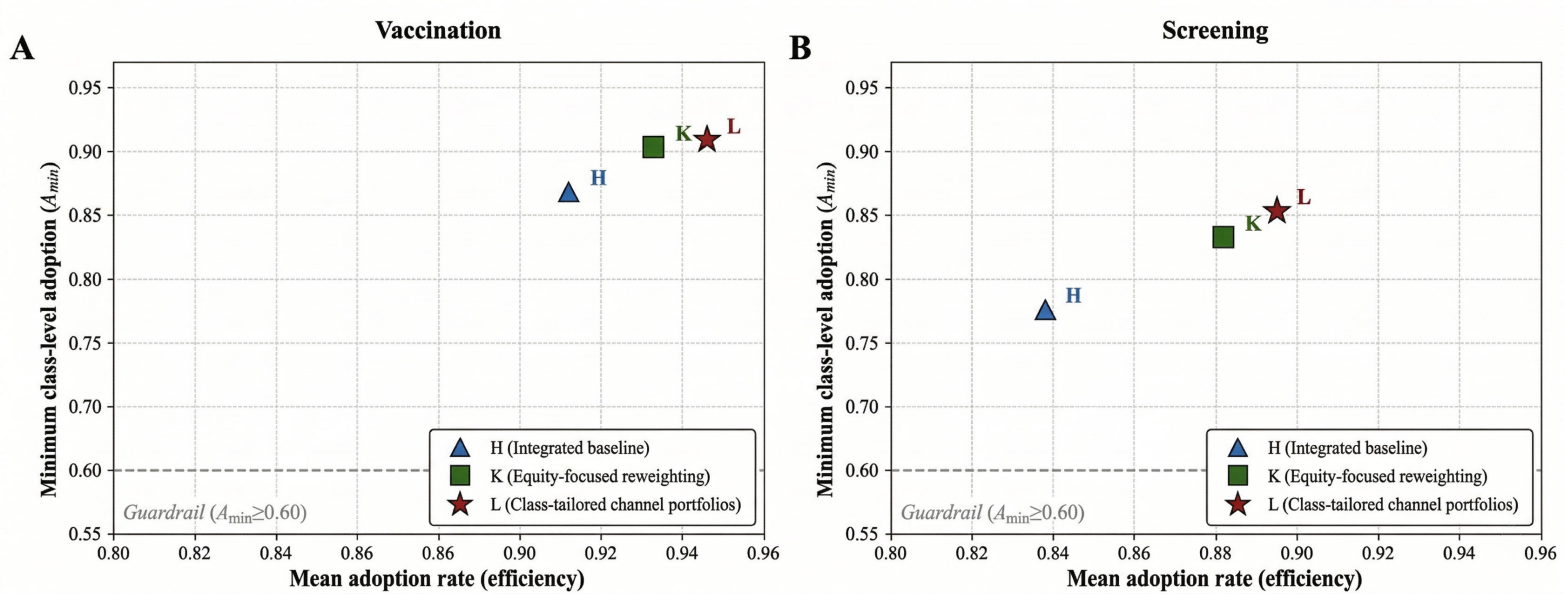
Efficiency-equity trade-offs for integrated baseline and personalized campaigns (H-L) for vaccination (A) and screening (B).

Table 3 summarizes the mean adoption and key equity metrics for scenarios H, K, and L. Figure 4 shows the resulting efficiency-equity trade-offs. Tables S1 and S2 in Multimedia Appendix 2 report the additional inequality indices and scenario-level adoption and equity metrics for all campaigns (A-O), including the TV-only campaign (B), low- and high-budget variants (I-J), and framing variants (M-O).

**Table 3.** Mean adoption and key equity metrics for scenarios H (all channels), K (equity-focused reweighting), and L (class-tailored channel portfolios).

Scenario	Mean adoption, n (%)	A_min_, %	90‐10 gap, percentage points
Vaccination (N=2405)			
H	2193 (91.2)	86.8	5.7
K	2244 (93.3)	90.3	4.5
L	2275 (94.6)	90.9	4.7
Screening (N=2400)			
H	2011 (83.8)	77.6	9.2
K	2117 (88.2)	83.2	7.4
L	2148 (89.5)	85.3	6.2

### Message Framing (RQ5)

Averaged across scenarios M to O, increasing loss framing intensity (k_neg_) produced modest, monotonic gains. For vaccination (N=2405), raising k_neg_ from 0.95 to 1.10 increased adoption from 92.3% (n=2220) to 92.9% (n=2234), a gain of about 14 adopters (0.6 percentage points). Mean time to adoption decreased from 5.62 (SD 0.16) to 5.54 (SD 0.17) months. For screening (N=2400), raising k_neg_ from 1.05 to 1.20 increased adoption from 88.8% (n=2131) to 89.7% (n=2153), a gain of about 22 adopters (0.9 percentage points). Mean time to adoption decreased from 6.01 (SD 0.16) to 5.93 (SD 0.18) months.

In the television-based screening scenario (M; N=2400), increasing k_neg_ from 1.05 to 1.20 increased adoption from 91.4% (n=2194) to 92.3% (n=2215), with parallel reductions in time to adoption. Digital-only screening (N) showed lower overall adoption but similar incremental gains across the same k_neg_ range. Gains between adjacent intensity levels were small (≤0.5 percentage points) and tended to taper at higher k_neg_. The 95% percentile intervals overlapped, and we observed no evidence of backfiring within the tested range. Detailed dose-response curves and class-level heterogeneity by scenario and target are reported in Table S3 and Figure S1 in Multimedia Appendix 3.

### Sensitivity Analyses

Using the prespecified stability benchmarks (ρ≥0.90 and Δrank≤1) as heuristics, scenario ordering for mean adoption was stable across prespecified axes (link function, weight allocation, reinforcement threshold, and network uncertainty). Maximum rank shifts were small (Δrank≤2), with the social-centric weight allocation variant for screening showing Δrank=2 while preserving the qualitative ordering. Full protocols and stability statistics are reported in Table S1 in Multimedia Appendix 3.

We changed the social reinforcement threshold from *τ*=2 (≥2 adopting neighbors) to *τ*=1 (≥1 adopting neighbor) and regenerated networks with alternative random seeds. These modifications left the scenario ordering unchanged for both tasks (ρ=1.00; Δrank=0). Alternative weight allocation variants produced only minor shifts for screening (ρ as low as 0.90; Δrank up to 2) while preserving the qualitative ordering. Comparisons between logistic and clipped-linear link functions preserved these rankings for mean adoption in both tasks.

## Discussion

### Principal Findings

Across the 15 prespecified scenarios, 5 patterns summarized how campaign design choices shaped efficiency and equity among older adults. These patterns were similar for vaccination and screening and remained stable across sensitivity analyses.

First, efficiency was driven mainly by channel allocation. The TV-only strategy (B) produced some of the highest mean adoption rates under a fixed per-step budget. Mixed-channel portfolios (E-H) improved outcomes relative to the background-only control (A) but did not surpass B, suggesting that dividing limited exposure across channels diluted reinforcement within high-TV–affinity clusters. Under fixed budgets, a single strong channel can therefore outperform intuitive “balanced mix” strategies.

Second, personalization shifted the portfolio toward a more favorable efficiency-equity trade-off within the modeled scenario space. The equity-focused reweighting intervention (K) produced modest gains in mean adoption but larger improvements in A_min_ and the 90‐10 gap by lifting the lowest-performing class. The class-tailored strategy (L) showed a strong combined profile under fixed budgets. For vaccination, it matched the efficiency of B while improving equity. For screening, it achieved adoption comparable to B with narrower disparities. Taken together, these results suggest that class-tailored allocations can improve equity without necessarily reducing effectiveness. Under the modeled assumptions, they may also yield simultaneous improvements relative to uniform integrated mixes.

Third, high-budget expansion (J) increased average adoption and narrowed disparities, but it required higher total exposure. In contrast, K and L met guardrail criteria while operating under fixed budgets, indicating that reallocating exposure can achieve equity gains without increasing total exposure.

Fourth, framing acted as a secondary adjustment rather than a primary lever. Stronger loss framing produced small, monotonic improvements (<1 percentage point) in both tasks and showed no evidence of backfiring within the tested range. However, these increments were minor compared with differences driven by channel allocation and personalization [45,46].

Finally, these strategic patterns were robust. Scenario orderings were stable across alternative reinforcement thresholds, weight allocations, and network realizations. Results under logistic versus clipped-linear link functions showed the same qualitative hierarchy.

### Comparison With Prior Work

These findings relate to 4 strands of prior work: equity-focused implementation frameworks, media campaign evaluations, personalization in digital health, and ABMs of preventive behaviors.

First, implementation science frameworks such as the PRECEDE-PROCEED model and RE-AIM/PRISM increasingly emphasize equity [13,16]. However, they offer limited operational guidance on how to allocate budgets across channels and segments under explicit constraints. Most evaluations still describe who was reached and how effects varied after implementation [15,17]. Our model instead uses equity metrics as prespecified guardrails that screen and flag campaign portfolios once equity thresholds are violated. In doing so, it treats equity as an ex ante decision rule for digital health strategic planning rather than a purely descriptive endpoint.

Second, media campaign studies usually examine single-channel effects or short-term digital engagement metrics. Television is known to be effective for older adults, and digital channels offer heterogeneous reach, but few studies compare fixed-budget channel portfolios [18,58,59]. Existing work rarely tests whether multisource exposure creates synergy or whether splitting limited resources across channels weakens reinforcement. Our findings show that, under complex contagion dynamics, mixed strategies at fixed budgets do not outperform a strong single-channel allocation, helping to clarify a long-standing ambiguity in the campaign literature.

Third, personalization is often promoted as a way to improve relevance and engagement, while many authors warn that tailoring may widen inequalities by favoring digitally advantaged groups [60,61]. Reviews document these risks but seldom compare different personalization philosophies under common efficiency and equity metrics [60,61]. By contrasting equity-focused reweighting (K) with class-tailored channel portfolios (L), we show that personalization need not exacerbate disparities. When designed to lift the lowest-performing class or align exposure with segment-specific media affinities, personalization can improve both mean adoption and distributional equity.

Fourth, most ABMs of preventive behaviors have focused on disease transmission or generic opinion dynamics [28,32]. Few models combine nationally representative microdata, latent class segmentation, multilayer networks, and prespecified equity constraints within a single portfolio-testing framework [28,32]. In this study, the ABM links empirical population heterogeneity with mechanistic diffusion dynamics, allowing planners to test channel mixes, reallocations, and framing intensities under consistent, reproducible conditions.

Taken together, these features define a budget-constrained, equity-aware decision framework for preventive campaigns for older adults. This framework helps explain why some channel and personalization strategies are more favorable than others on both efficiency and equity within the modeled scenario space.

### Implications for Digital Health Strategic Planning

These results are intended to support comparative decision-making for campaign design rather than point forecasting. Planners can use the model to compare candidate portfolios that vary channel mix and targeting under shared budget and equity constraints. To operationalize the scenario inputs, the relative exposure weights can be interpreted as the intended distribution of campaign effort across channels, and these weights can be translated into proportional budget shares or delivery targets using standard planning metrics (eg, television reach and frequency; digital reach and impressions; print distribution volume). The outputs provide comparative evidence for selecting among portfolios, with efficiency and equity evaluated jointly using the study’s equity metrics and guardrails.

These findings suggest 3 priorities for public health planners working under budget constraints.

First, channel allocation should be treated as the primary strategic lever. Across both vaccination and screening, TV-only allocations produced some of the highest mean adoption levels under fixed budgets. Mixed portfolios improved outcomes but diluted reinforcement compared with television alone. For older adult populations with strong offline clustering, concentrating resources on a high-reach channel may therefore be more effective than spreading budgets thinly across multiple platforms.

Second, equity-oriented reallocation is both feasible and effective. The equity-focused reweighting strategy (K) raised minimum class-level adoption and narrowed class disparities with only small changes in mean outcomes. This shows that modest, budget-neutral shifts can materially improve equity. The class-tailored strategy (L) further improved both mean and distributional outcomes, indicating that segmentation-based resource allocation need not exacerbate inequities. These results suggest that agencies with basic segmentation data and media-buying capacity could adopt class-tailored portfolios to achieve efficiency-equity gains without increasing total exposure budgets.

Third, loss framing yielded only minor incremental gains within the tested range, so it should follow—rather than replace—allocation and targeting decisions.

Together, these implications position equity guardrails as a practical planning benchmark. Planners can start from an integrated channel mix and deprioritize strategies that fall below minimum subgroup adoption or widen disparities. Among the remaining options, they can consider conservative reallocations or class-tailored portfolios—such as scenarios K and L— that show a more favorable efficiency-equity trade-off while maintaining strong mean adoption.

### Limitations

Several limitations should be noted.

First, the ABM relied on structural assumptions about media influence, baseline propensity, and network topology. These assumptions simplify complex behavioral processes and may omit unmeasured factors. Calibration matched survey targets, and scenario rankings were stable across sensitivity checks. However, some model misspecification may remain.

Second, offline and online networks were approximated using survey-derived degree targets and a Watts-Strogatz small-world structure. Real-world networks may differ in clustering, multiplexity, tie strength, and mixing. These approximations may overestimate or underestimate the strength of social influence. They may also distort within-group versus between-group reinforcement. Rank stability across alternative network realizations suggests that the qualitative ordering of strategies is unlikely to depend on a single specification. However, more detailed network data could refine these structures. We did not test alternative class structures, and the sensitivity of scenario rankings to segmentation choices remains a topic for future work. Segmentation was cross-sectional, so we could not test temporal stability or class transitions.

Third, the model focused on exposure and diffusion rather than message content and quality, source trustworthiness, or system-level access barriers. Reactance, stigma, and message fatigue were not modeled, and strong fear-based or stigmatizing loss frames fell outside the tested k_neg_ range. Modest loss framing did not show evidence of backfiring within the simulated range, but these results should not be generalized to more aggressive messaging strategies.

Fourth, the model evaluated a 1-year campaign horizon and did not capture longer-term maintenance, multiyear cycles, or downstream health outcomes. Adoption was modeled as a single-step absorbing behavior, whereas real-world screening and vaccination involve repeated decisions and system-level interactions. Budget was represented as relative exposure intensity rather than monetary spending. Therefore, the results do not directly translate to monetary costs or cost patterns across media markets.

Finally, the analysis is based on matched Korean survey data. Several contextual features—such as high television reach, strong offline clustering, and a pronounced digital divide—may be specific to this setting. Applying the framework elsewhere would likely require recalibrating context-dependent inputs, such as channel reach patterns, the magnitude of the digital divide, and network mixing or clustering. In contrast, the model’s core mechanisms—heterogeneous media affinity and reinforcement-based diffusion through social ties—may be more portable. Their magnitudes, however, may vary across settings. External validation in additional settings will be important for assessing transportability and for refining model-based decision tools.

### Conclusions

This study used a data-driven ABM to examine how channel allocation, personalization, and loss framing shape preventive campaign performance for older adults under fixed budgets. In this survey-calibrated simulation, equity-oriented personalization of television and digital exposure improved subgroup equity and increased mean adoption relative to uniform integrated mixes and simple channel diversification. We evaluated scenarios using prespecified, illustrative equity guardrails. These patterns suggest that accounting for diffusion heterogeneity and reinforcement can help identify portfolios that strengthen equity without necessarily reducing overall performance.

For practice, an integrated channel mix can serve as a pragmatic starting point. Equity guardrails can then help identify portfolios that erode minimum subgroup adoption or widen disparities. Among the strategies that pass these guardrails, conservative reallocations can offer budget-neutral equity gains. Class-tailored portfolios may provide a more favorable efficiency-equity profile where segmentation and media-buying capacity are available. These conclusions apply primarily to settings with pronounced media heterogeneity and digital divides. They should be interpreted in light of the model’s structural assumptions, 1-year horizon, and reliance on matched Korean survey data. Future work should validate these strategic patterns in other health systems and link simulated diffusion to observed exposure and engagement in real campaigns. It should also extend guardrail-based planning tools to richer communication, organizational, and multiyear decision contexts.

## Supplementary material

10.2196/88429 Multimedia Appendix 1 Additional methods.

10.2196/88429 Multimedia Appendix 2 Scenario-level adoption and equity metrics.

10.2196/88429 Multimedia Appendix 3 Sensitivity and robustness.

10.2196/88429 Multimedia Appendix 4 Calibration and validation diagnostics.

## Abbreviations

ABM: agent-based model
AIC: Akaike information criterion
BIC: Bayesian information criterion
KMP: Korea Media Panel Survey
KNHANES: Korea National Health and Nutrition Examination Survey
PI: percentile interval
PRISM: Practical Robust Implementation and Sustainability Model
PROGRESS-Plus: place of residence; race, ethnicity, culture, and language; occupation; gender and sex; religion; education; socioeconomic status; and social capital—plus additional equity-relevant factors
RDI: relative disparity index
RE-AIM: Reach, Effectiveness, Adoption, Implementation and Maintenance
RQ: research question
TV: television
