# Supplementary material for: Channel Allocation and Equity in Preventive Campaigns for Older Adults: Agent-Based Modeling Study
Source: J Med Internet Res. 2026 Apr 1;28:e88429. doi: 10.2196/88429 (PMC13041628; doi:10.2196/88429)
Supplement: Multimedia Appendix 1 [file jmir-v28-e88429-s001.docx]

## Multimedia Appendix 1. Additional methods

This appendix provides additional details on the statistical matching procedure, latent class analysis (LCA), and agent initialization referenced in the Methods section.

### A. Statistical matching covariates and diagnostics

We linked KNHANES 2022 recipients to KMP 2022 donors using one-to-one nearest-neighbor matching with replacement based on Gower distance. Matching covariates were selected to capture key sociodemographic and household characteristics that may influence media use, digital access, social connectedness, and preventive behaviors. Covariates used for statistical matching were:

- education
- occupation group
- sex
- age
- region
- household size
- household composition
- housing type
- marital status

We assessed match quality using the distribution of Gower distances across all matched pairs. Among 2,405 matched pairs, the mean distance was 0.0283, the median was 0.0219, and the 95th percentile was 0.0689. Most pairs (2,399/2,405, 99.8%) had distances ≤0.10; only 6 pairs fell between 0.10 and 0.20, and none exceeded 0.20. We applied a prespecified acceptance threshold of ≤0.20 for all pairs, and the observed distance distribution met this requirement.

### B. Latent class analysis indicators and model selection

We applied LCA to the matched dataset to identify segments of older adults with distinct media, digital, social, and preventive profiles. Indicator domains were chosen to reflect core sociodemographic, behavioral, and structural characteristics:

- sociodemographics (eg, age, education)
- digital literacy (eg, search skill, messaging proficiency)
- media use (eg, TV viewing time, digital news pathways, over-the-top [OTT] streaming frequency)
- social activity (eg, friendship contact frequency, offline meeting patterns)
- health-behavior anchors (influenza vaccination and routine health screening)

We discretized continuous indicators into ordinal categories using prespecified binning rules such as tertiles or quantiles. Estimation settings were as follows:

- models were estimated via the EM algorithm using the glca package in R
- we ran 100 random initializations and retained the 20 best for restart optimization
- convergence was defined as an absolute change in log-likelihood < 1×10⁻⁶ under a fixed random seed

We estimated models with K = 3–10 latent classes and compared them using the Bayesian Information Criterion (BIC), entropy, and the bootstrap likelihood ratio test (BLRT). The six-class solution (K = 6) minimized BIC (86,578.57), achieved high entropy (approximately 0.92), and showed significant BLRT improvements over adjacent solutions, indicating a parsimonious and well-separated segmentation. For descriptive summaries, we assigned each individual to their modal class. For simulation and personalization, we retained each individual’s full posterior probability vector and used these probabilities as class-based personalization weights. Full model-fit statistics for all K values are reported in Table S1.

Table S1. Latent class model fit statistics for models with 3–10 classes.

| K | Npar | log-likelihood | AIC | BIC | Entropy | df | G²  (vs saturated) | BLRT  Δ deviance (vs K–1) | BLRT  p-value |
| --- | --- | --- | --- | --- | --- | --- | --- | --- | --- |
| 3 | 182 | -43361.60 | 87087.20 | 88140.12 | 0.88 | 2222 | 49378.83 | — | — |
| 4 | 243 | -42658.64 | 85803.28 | 87209.11 | 0.91 | 2161 | 47972.91 | 1405.92 | <0.001 |
| 5 | 304 | -42265.49 | 85138.99 | 86897.72 | 0.91 | 2100 | 47186.62 | 786.29 | <0.001 |
| 6 | 365 | -41868.47 | 84466.93 | 86578.57 | 0.92 | 2039 | 46392.57 | 794.06 | <0.001 |
| 7 | 426 | -41657.68 | 84167.36 | 86631.90 | 0.91 | 1978 | 45970.99 | 421.57 | <0.001 |
| 8 | 487 | -41476.76 | 83927.51 | 86744.96 | 0.91 | 1917 | 45609.15 | 361.85 | <0.001 |
| 9 | 548 | -41360.03 | 83816.06 | 86986.40 | 0.90 | 1856 | 45375.69 | 233.46 | <0.001 |
| 10 | 609 | -41212.49 | 83642.98 | 87166.23 | 0.89 | 1795 | 45080.61 | 295.08 | <0.001 |

### C. Agent initialization and degree priors

Each matched individual was instantiated as an agent with a channel-affinity vector and an offline degree prior derived from survey responses. Channel affinities distinguished between digital and traditional channels and were constructed from self-reported media-use behaviors:

- digital search behavior
- instant messaging use
- OTT streaming frequency
- television viewing time
- newspaper reading time

We also derived an offline degree prior for each agent based on self-reported social-activity frequency. Responses were grouped into five ordered levels and mapped to target degrees of 3, 4, 6, 8, and 12, so that agents reporting more frequent offline activity received higher degree targets. During network construction, each agent’s offline degree prior served as the target degree in a degree-targeted graph-generation algorithm for the offline layer, which approximated these targets while avoiding self-loops and multi-edges. The online layer was generated separately as a Watts–Strogatz small-world network with fixed mean degree and rewiring probability, independent of offline degree targets.
