# Supplementary material for: Channel Allocation and Equity in Preventive Campaigns for Older Adults: Agent-Based Modeling Study
Source: J Med Internet Res. 2026 Apr 1;28:e88429. doi: 10.2196/88429 (PMC13041628; doi:10.2196/88429)
Supplement: Multimedia Appendix 2 [file jmir-v28-e88429-s002.docx]

## Multimedia Appendix 2. Scenario-level adoption and equity metrics

All scenario-level values in these tables reflect the primary full-population analysis (vaccination N=2,405; screening N=2,400; 100 Monte Carlo replications). Adoption rate, A_min_, and the 90–10 gap are reported as proportions (0–1). Sensitivity analyses based on an 800-agent subsample are reported separately in Multimedia Appendix 4.

Table S1. Vaccination: Scenario-level adoption and equity metrics for campaigns A–O.

| scenario | Adoption rate (mean) | A_min_ | 90–10 gap | RDI | Atkinson A_0.5_ | Theil T |
| --- | --- | --- | --- | --- | --- | --- |
| A | 0.8493 | 0.7873 | 0.0756 | 1.1346 | 0.0005 | 0.0010 |
| B | 0.9418 | 0.8783 | 0.0636 | 1.1075 | 0.0003 | 0.0006 |
| C | 0.9076 | 0.8601 | 0.0708 | 1.1062 | 0.0003 | 0.0007 |
| D | 0.8572 | 0.8003 | 0.0711 | 1.1249 | 0.0004 | 0.0008 |
| E | 0.9301 | 0.8911 | 0.0524 | 1.0808 | 0.0002 | 0.0004 |
| F | 0.9126 | 0.8525 | 0.0662 | 1.1132 | 0.0004 | 0.0007 |
| G | 0.8878 | 0.8453 | 0.0611 | 1.0969 | 0.0003 | 0.0005 |
| H | 0.9121 | 0.8681 | 0.0574 | 1.0907 | 0.0002 | 0.0005 |
| I | 0.8987 | 0.8468 | 0.0623 | 1.1048 | 0.0003 | 0.0006 |
| J | 0.9918 | 0.9802 | 0.0159 | 1.0203 | 0.0000 | 0.0000 |
| K | 0.9330 | 0.9030 | 0.0447 | 1.0660 | 0.0001 | 0.0003 |
| L | 0.9457 | 0.9088 | 0.0467 | 1.0695 | 0.0001 | 0.0003 |
| M | 0.9436 | 0.8826 | 0.0619 | 1.1042 | 0.0003 | 0.0006 |
| N | 0.9081 | 0.8604 | 0.0720 | 1.1080 | 0.0003 | 0.0007 |
| O | 0.9323 | 0.8939 | 0.0511 | 1.0779 | 0.0002 | 0.0004 |

Table S2. Screening: Scenario-level adoption and equity metrics for campaigns A–O.

| scenario | Adoption rate (mean) | A_min_ | 90–10 gap | RDI | Atkinson A_0.5_ | Theil T |
| --- | --- | --- | --- | --- | --- | --- |
| A | 0.7449 | 0.6833 | 0.0912 | 1.1813 | 0.0009 | 0.0017 |
| B | 0.8936 | 0.8501 | 0.0639 | 1.0985 | 0.0003 | 0.0006 |
| C | 0.8292 | 0.7260 | 0.1539 | 1.2662 | 0.0018 | 0.0036 |
| D | 0.7570 | 0.6980 | 0.0898 | 1.1754 | 0.0008 | 0.0016 |
| E | 0.8684 | 0.8095 | 0.0864 | 1.1431 | 0.0005 | 0.0011 |
| F | 0.8407 | 0.7936 | 0.0711 | 1.1211 | 0.0004 | 0.0008 |
| G | 0.7990 | 0.7137 | 0.1257 | 1.2295 | 0.0013 | 0.0027 |
| H | 0.8376 | 0.7755 | 0.0915 | 1.1587 | 0.0007 | 0.0013 |
| I | 0.8144 | 0.7490 | 0.0986 | 1.1760 | 0.0008 | 0.0016 |
| J | 0.9772 | 0.9492 | 0.0378 | 1.0520 | 0.0001 | 0.0002 |
| K | 0.8817 | 0.8316 | 0.0738 | 1.1147 | 0.0004 | 0.0008 |
| L | 0.8952 | 0.8527 | 0.0617 | 1.0968 | 0.0003 | 0.0005 |
| M | 0.9020 | 0.8583 | 0.0649 | 1.1005 | 0.0003 | 0.0006 |
| N | 0.8313 | 0.7175 | 0.1695 | 1.2953 | 0.0022 | 0.0043 |
| O | 0.8762 | 0.8153 | 0.0899 | 1.1471 | 0.0006 | 0.0012 |

Table S3. Latent class profiles and baseline preventive uptake.^a^

| Variable | Class 1 (n=466) | Class 2 (n=245) | Class 3 (n=413) | Class 4 (n=438) | Class 5 (n=534) | Class 6 (n=309) |
| --- | --- | --- | --- | --- | --- | --- |
| Gender | Female (92%) | Female (82%) | Male (88%) | Female (82%) | Balanced  (Female 52%, Male 48%) | Male (75%) |
| Age | ≥76 years (89%) | ≤80 years (85%) | ≤80 years (95%) | ≤80 years (95%) | No dominant age group | 65–70 years (87%) |
| Household composition | Mostly single-person households (58%) | Single-person (67%); two-generation with children (20%) | Couple-only households (72%) | Couple-only (61%); two-generation (33%) | Couple-only households (76%) | Couple-only (38%); two-generation (51%) |
| Education | Primary school or less (85%) | Evenly distributed; college+ (3%) | Evenly distributed; college+ (6%) | Evenly distributed; college+ (4%) | Mostly primary or middle school or less | Mostly high school (63%) and college+ (23%) |
| Spouse | No spouse (99%) | Spouse present (100%) | Spouse present (96%) | Spouse present (99%) | Spouse present (99%) | Spouse present (87%) |
| Monthly personal income | ≤1 million KRW (95%) | 1.01–2 million KRW (66%) and below | 1.01–2 million KRW (80%) and some higher | <0.5 million KRW (92%) | ≤1 million KRW (86%) | Higher income; ≥1.01 million KRW (93%), ≥3 million KRW (27%) |
| Occupation | Not working (85%) | Not working (55%), agricultural and manual labor jobs (40%) | Not working (25%), mainly manual labor and agricultural jobs | Not working (86%) | Not working (72%) | Lowest non-working share (10%); many service/sales and craft/machine jobs |
| Instant messaging ability | Cannot send instant messages (84%) | Can send (63%) | Can send (71%) | Can send (80%) | Cannot send (70%) | Highly proficient; can send (93%) |
| Search engine ability | Cannot search (96%) | Mostly low to moderate ability | No dominant pattern | Many with positive ability (about 38%) | Cannot search (90%) | Can search (82%) |
| Fact-checking tendency | Rarely checks whether information is true (56%) | Mostly neutral or negative about checking | Mostly neutral or low tendency to check | Mostly neutral or low tendency to check | Mainly negative responses | More likely to check; neutral to positive responses (about 70%) |
| Newspaper medium | Does not read newspapers (99%) | Mostly none (74%); some via smartphone app (24%) | Smartphone app (46%); none (43%) | Half none, half smartphone app | Does not read newspapers (96%) | Lowest 'none' share (19%); mostly smartphone app (60%) |
| Daily time on print media | Almost none (0 minutes for 93%) | Almost none (0 minutes for 91%) | Almost none (0 minutes for 89%) | Almost none (0 minutes for 88%) | Almost none (0 minutes for 87%) | Almost none (0 minutes for 86%) |
| Daily TV viewing time | ≥4 hours (81%) | ≥4 hours (72%) | 2.5–6 hours (66%) | ≥4 hours (77%) | ≥4 hours (73%) | Less TV; about 1–4 hours (70%) |
| Social activity frequency | No clear dominant pattern (generally low) | 1–3 times per month (52%) | 1–3 times per month (56%) | 1–3 times per month (46%); once every 3–6 months (30%) | Generally low social activity | 1–3 times per month (about 50%) |
| Daily smartphone use | ≤1 hour (94%) | 1 minute–2 hours (78%) | 1 minute–2 hours (77%) | 1 minute–2 hours (76%) | 0 hours (55%) | 0 hours (0%), 1 minute–2 hours (58%), >2 hours (42%) |
| OTT service use | Nonresponse/does not use (99%) | About half nonresponse; others spread across levels | Many nonresponse; some 1–4 times per week | 1–4 times per week (31%); almost daily (20%) | Nonresponse/does not use (97%) | Lowest nonresponse; many monthly or almost-daily users |
| Screening uptake | Yes (66%) | Yes (77%) | Yes (74%) | Yes (82%) | Yes (71%) | Yes (78%) |
| Influenza vaccination uptake | Yes (85%) | Yes (86%) | Yes (87%) | Yes (80%) | Yes (88%) | Yes (63%) |

A Percentages indicate within-class proportions; denominators are the class sizes shown in the table.
