# Supplementary material for: Channel Allocation and Equity in Preventive Campaigns for Older Adults: Agent-Based Modeling Study
Source: J Med Internet Res. 2026 Apr 1;28:e88429. doi: 10.2196/88429 (PMC13041628; doi:10.2196/88429)

## Multimedia Appendix 3. Sensitivity and robustness

This appendix reports the protocol and diagnostics used to assess the structural robustness of the scenario-level efficiency–equity conclusions. We varied four modeling axes and evaluated whether the scenario ranking by final adoption (mean adoption at month 12) remained stable across sensitivity configurations.

### A. Sensitivity Axes

We examined four prespecified axes of model variation:

- Link function (logistic vs clipped-linear)
- Weight allocation (baseline, balanced, media-centric, social-centric)
- Social-reinforcement threshold (τ = 2 vs τ = 1)
- Network uncertainty (two independently generated hybrid networks)

These axes tested robustness to core structural assumptions about diffusion, social reinforcement, and contact patterns.

### B. Simulation Protocol

- We simulated each sensitivity configuration using 100 Monte Carlo replications on an 800-agent subsample drawn from the full analytic population.
- For the link-function sensitivity axis, we reran all scenarios under both link-function specifications (logistic and clipped-linear), holding all other settings fixed.
- For network-uncertainty tests, we regenerated both offline and online layers under two independent seeds while preserving the same marginal degree targets.
- Additional full-population (N = 2,405) runs were used to verify that scenario rankings obtained from the subsample generalized to the full network.
- For each configuration, we computed a 15-element vector of final adoption rates (one per scenario) and compared its ranking with the baseline ordering. For loss-framing scenarios (M–O), we averaged final adoption over the tested k_neg_ levels when constructing the scenario-level vector.

### C. Rank‑Stability Metrics

We summarized robustness using two descriptive metrics:

- Spearman rank correlation (ρ): correlation between baseline and sensitivity scenario-level adoption vectors.
- Maximum rank shift (Δrank): largest absolute difference in rank for any scenario between the two orderings.

Following common ABM practice, we treated ρ ≥ 0.90 and Δrank ≤ 1 as heuristic benchmarks for high stability. We also report the maximum observed rank shifts, which reached Δrank = 2 for the social-centric weight-allocation variant in screening while preserving the qualitative ordering.

### D. Global Sensitivity Results

Across all axes, scenario-level rankings were highly consistent with the baseline ordering. Spearman correlations ranged from approximately 0.90 to 1.00, with most ≥0.98, and maximum rank shifts were small (Δrank ≤ 2). Detailed stability statistics for all axes appear in Table S4-1.

- Weight allocation: Balanced and media-centric variants yielded ρ ≥ 0.98 with Δrank ≤ 1. Social-centric screening showed modest perturbation (ρ ≈ 0.90–0.98; Δrank up to 2).
- Social-reinforcement threshold: Lowering τ from 2 to 1 produced identical rankings for vaccination and only negligible variation for screening.
- Network uncertainty: Independent hybrid networks generated under alternative random seeds reproduced the exact baseline rankings (ρ = 1.00; Δrank = 0).
- Link function: Logistic and clipped-linear specifications produced nearly identical rankings for both vaccination and screening (ρ = 0.99–1.00; Δrank ≤ 1).

These findings suggest that the scenario hierarchy is largely shaped by the modeled diffusion dynamics and is not driven by any single modeling choice.

Table S1. Rank stability across sensitivity variants.

| Target | Sensitivity axis | Variants | Spearman ρ (range)^a^ | Max Δrank^b^ |
| --- | --- | --- | --- | --- |
| Vaccination | Weight allocation | Baseline, balanced, media-centric, social-centric | 0.98–1.00 | 0–1 |
| Vaccination | Social-reinforcement threshold | τ = 1 vs τ = 2 | 1.00–1.00 | 0–0 |
| Vaccination | Network uncertainty | Hybrid network realizations (alternative seeds) | 1.00–1.00 | 0–0 |
| Vaccination | Link function | Logistic vs clipped-linear | 0.99–0.99 | 1–1 |
| Screening | Weight allocation | Baseline, balanced, media-centric, social-centric | 0.90–1.00 | 0–2 |
| Screening | Social-reinforcement threshold | τ = 1 vs τ = 2 | 1.00–1.00 | 0–0 |
| Screening | Network uncertainty | Hybrid network realizations (alternative seeds) | 1.00–1.00 | 0–0 |
| Screening | Link function | Logistic vs clipped-linear | 1.00–1.00 | 0–0 |

^a^ Spearman rank correlations (ρ) comparing each sensitivity configuration with the baseline scenario ranking.

^b^ Maximum absolute rank shift (Δrank) relative to the baseline scenario ranking.

### E. Scenario K sensitivity to reweighting intensity (δ = 0.02 vs δ = 0.05).

We examined whether the strength of the equity-focused reweighting in scenario K altered efficiency–equity conclusions. We compared a higher-intensity boost (δ = 0.05) with the main-text setting (δ = 0.02) while holding all other parameters fixed, using the same 800-agent subsample as in other sensitivity tests. Increasing δ produced only small changes in mean adoption and equity metrics for both vaccination and screening (Table S4-2). The qualitative conclusions were unchanged: the more conservative reweighting configuration (δ = 0.02) remained preferable because it preserved mean adoption while slightly improving A_min_ and the 90–10 gap relative to δ = 0.05.

Table S2. Scenario K sensitivity to reweighting intensity (δ = 0.02 vs 0.05).

| Target | δ | Mean adoption (proportion) | A_min_ (proportion) | 90–10 gap (proportion) |
| --- | --- | --- | --- | --- |
| Vaccination | 0.02 | 0.931 | 0.921 | 0.019 |
| Vaccination | 0.05 | 0.930 | 0.917 | 0.029 |
| Screening | 0.02 | 0.901 | 0.875 | 0.037 |
| Screening | 0.05 | 0.899 | 0.869 | 0.046 |

### F. Loss-Framing Dose–Response Analysis (scenarios M–O)

We examined dose–response patterns for loss-framed messaging by varying framing intensity (k_neg_) for scenarios M–O, separately for vaccination and screening. For each target–scenario–intensity combination, we report mean adoption, 95% percentile intervals, and mean time to adoption with corresponding uncertainty intervals (Table S4-3).

Across all scenarios, increasing k_neg_ produced modest, monotonic improvements in adoption and slightly shorter times to adoption. Adjacent intensity levels typically differed by less than 0.5 percentage points, and uncertainty intervals overlapped considerably. These patterns support the main-text conclusion that loss framing acts as a secondary tuning lever rather than a primary driver of adoption dynamics. Figure S4 presents the same results as dose–response curves.

Table S3. Adoption outcomes and time-to-adoption by loss-framing intensity (k_neg_) for scenarios M–O.

| Target | Scenario | Framing intensity (k_neg_) | Adoption rate (mean, proportion) | Adoption rate (2.5th percentile, proportion) | Adoption rate (97.5th percentile, proportion) | Time to adoption, months (mean) | Time to adoption, months (2.5th percentile) | Time to adoption, months (97.5th percentile) |
| --- | --- | --- | --- | --- | --- | --- | --- | --- |
| Vaccination | M | 0.95 | 0.939 | 0.928 | 0.948 | 5.446 | 5.34 | 5.547 |
| Vaccination | M | 1.0 | 0.943 | 0.935 | 0.951 | 5.412 | 5.311 | 5.541 |
| Vaccination | M | 1.05 | 0.945 | 0.936 | 0.954 | 5.378 | 5.266 | 5.502 |
| Vaccination | M | 1.1 | 0.947 | 0.938 | 0.954 | 5.343 | 5.226 | 5.46 |
| Vaccination | N | 0.95 | 0.903 | 0.89 | 0.915 | 5.802 | 5.676 | 5.921 |
| Vaccination | N | 1.0 | 0.904 | 0.894 | 0.916 | 5.78 | 5.676 | 5.891 |
| Vaccination | N | 1.05 | 0.906 | 0.892 | 0.917 | 5.762 | 5.642 | 5.904 |
| Vaccination | N | 1.1 | 0.907 | 0.894 | 0.921 | 5.744 | 5.616 | 5.869 |
| Vaccination | O | 0.95 | 0.926 | 0.917 | 0.937 | 5.613 | 5.521 | 5.749 |
| Vaccination | O | 1.0 | 0.929 | 0.919 | 0.938 | 5.588 | 5.474 | 5.726 |
| Vaccination | O | 1.05 | 0.931 | 0.921 | 0.94 | 5.556 | 5.437 | 5.687 |
| Vaccination | O | 1.1 | 0.934 | 0.926 | 0.945 | 5.54 | 5.428 | 5.639 |
| Screening | M | 1.05 | 0.914 | 0.903 | 0.924 | 5.832 | 5.705 | 5.949 |
| Screening | M | 1.1 | 0.917 | 0.905 | 0.927 | 5.799 | 5.666 | 5.895 |
| Screening | M | 1.15 | 0.919 | 0.906 | 0.93 | 5.751 | 5.63 | 5.863 |
| Screening | M | 1.2 | 0.923 | 0.911 | 0.934 | 5.722 | 5.612 | 5.818 |
| Screening | N | 1.05 | 0.857 | 0.844 | 0.87 | 6.2 | 6.075 | 6.343 |
| Screening | N | 1.1 | 0.86 | 0.846 | 0.875 | 6.176 | 6.064 | 6.307 |
| Screening | N | 1.15 | 0.862 | 0.845 | 0.876 | 6.158 | 6.059 | 6.269 |
| Screening | N | 1.2 | 0.864 | 0.848 | 0.876 | 6.131 | 6.023 | 6.265 |
| Screening | O | 1.05 | 0.893 | 0.879 | 0.905 | 6.004 | 5.871 | 6.121 |
| Screening | O | 1.1 | 0.896 | 0.883 | 0.905 | 5.988 | 5.858 | 6.113 |
| Screening | O | 1.15 | 0.899 | 0.887 | 0.909 | 5.96 | 5.845 | 6.085 |
| Screening | O | 1.2 | 0.902 | 0.891 | 0.913 | 5.923 | 5.807 | 6.032 |

Figure S1. Dose–response curves for loss-framing intensity (k_neg_) by scenario (M–O) and target.


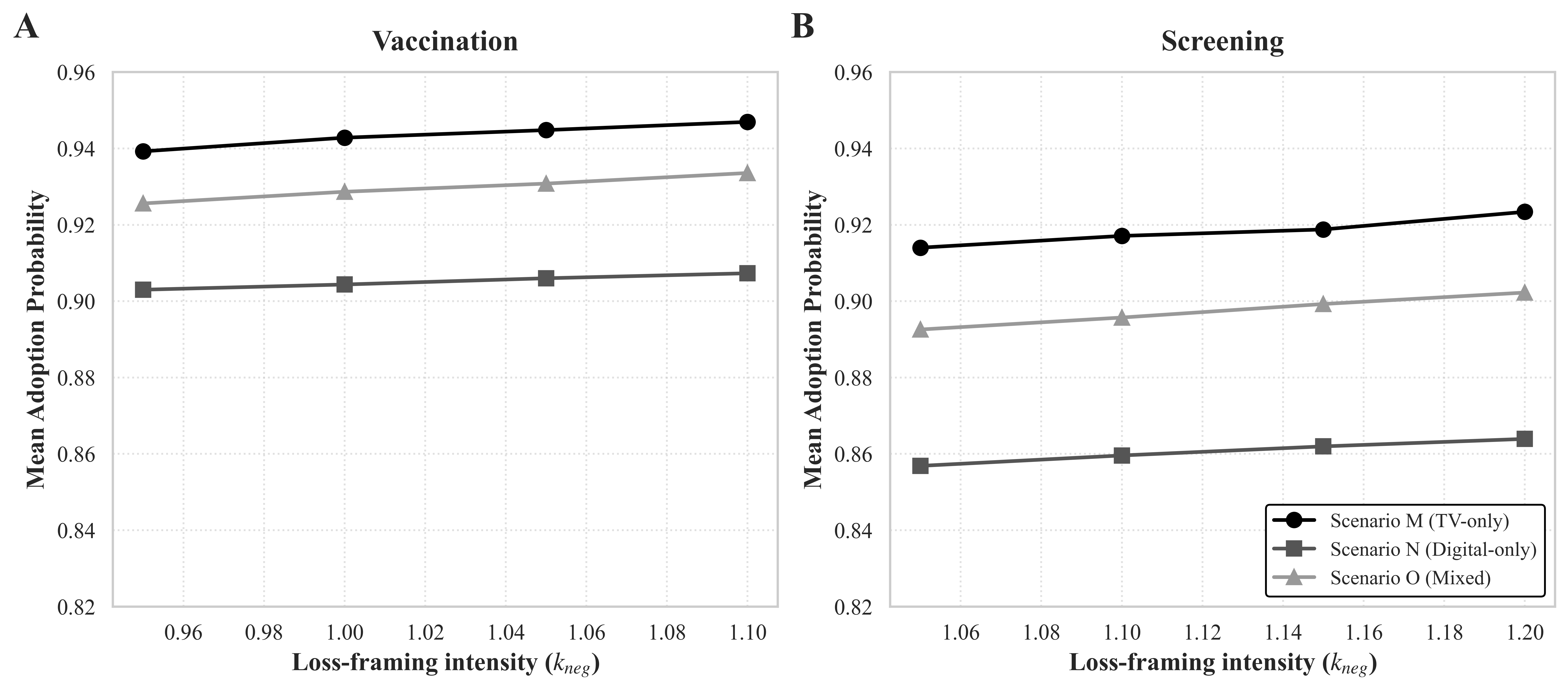

Supplement: Multimedia Appendix 3 [file jmir-v28-e88429-s003.docx]
