# Supplementary material for: Channel Allocation and Equity in Preventive Campaigns for Older Adults: Agent-Based Modeling Study
Source: J Med Internet Res. 2026 Apr 1;28:e88429. doi: 10.2196/88429 (PMC13041628; doi:10.2196/88429)
Supplement: Multimedia Appendix 4 [file jmir-v28-e88429-s004.docx]

## Multimedia Appendix 4. Calibration and validation diagnostics

### A. Calibration Procedure

Calibration was performed separately for vaccination and screening. For screening calibration and validation diagnostics, we excluded five participants with nonresponse (N=2,400). We first ran a grid search that varied the baseline propensity multiplier α from 0.80 to 1.20 in 0.05 increments and the background exposure WBG from 0.10 to 0.20. For each (α, WBG) pair, we simulated the background-only control scenario (A) 100 times on an 800-agent subsample and computed the absolute deviation from the KNHANES 2022 targets (0.848 for vaccination and 0.744 for screening). For screening, we then performed a second-stage refinement that varied α in 0.01 increments in a narrow band around the best coarse-grid value (α = 1.00), holding WBG fixed at 0.10. We selected the final screening parameter (α = 1.03) from the fine-grid candidates as the value that minimized the absolute deviation from the empirical target (0.744).

### B. Best-fitting Calibration Parameters

Table S1. Best-fitting calibration parameter sets for vaccination and screening under the background-only scenario.

| Task | α | W_BG_ | Mean adoption (proportion) | Target (proportion) | Absolute error^a^ |
| --- | --- | --- | --- | --- | --- |
| Vaccination | 1.15 | 0.12 | 0.848 | 0.848 | 0.0004 |
| Screening | 1.03 | 0.10 | 0.745 | 0.744 | 0.0009 |

^a^ Absolute error is the absolute difference between the simulated mean adoption under the calibrated background-only scenario and the KNHANES 2022 target for each task.

### C. Per-Class Calibration Results

Table S2. Observed and predicted adoption rates by latent class under the calibrated background-only scenario.

| Target | Class | Observed adoption (proportion) | Predicted adoption (proportion) | Absolute difference |
| --- | --- | --- | --- | --- |
| **Vaccination** |  |  |  |  |
|  | 1 | 0.850 | 0.872 | 0.022 |
|  | 2 | 0.824 | 0.862 | 0.037 |
|  | 3 | 0.869 | 0.873 | 0.003 |
|  | 4 | 0.797 | 0.851 | 0.055 |
|  | 5 | 0.876 | 0.878 | 0.001 |
|  | 6 | 0.625 | 0.763 | 0.138 |
| **Screening** |  |  |  |  |
|  | 1 | 0.665 | 0.700 | 0.035 |
|  | 2 | 0.763 | 0.782 | 0.019 |
|  | 3 | 0.738 | 0.761 | 0.022 |
|  | 4 | 0.826 | 0.808 | 0.019 |
|  | 5 | 0.721 | 0.748 | 0.027 |
|  | 6 | 0.779 | 0.785 | 0.006 |
